# Antispasmodic and vasodilator activities of *Morinda citrifolia *root extract are mediated through blockade of voltage dependent calcium channels

**DOI:** 10.1186/1472-6882-10-2

**Published:** 2010-01-13

**Authors:** Anwarul Hassan Gilani, Saf-ur-Rehman Mandukhail, Javeid Iqbal, Masoom Yasinzai, Nauman Aziz, Aslam Khan, Najeeb-ur-Rehman

**Affiliations:** 1 Natural Product Research Division, Department of Biological and Biomedical Sciences, Aga Khan University Medical College, stadium road, Karachi-74800, Pakistan; 2 Departments of Pharmacy and Biochemistry, University of Balochistan, Sariab road, Quetta, Pakistan; 3 King Saud University, Riyadh, Kingdom of Saudi Arabia

## Abstract

**Background:**

*Morinda citrifolia *(Noni) is an edible plant with wide range of medicinal uses. It occurs exclusively in tropical climate zone from India through Southeast Asia and Australia to Eastern Polynesia and Hawaii. The objective of this study was to explore the possible mode(s) of action for its antispasmodic, vasodilator and cardio-suppressant effects to rationalize its medicinal use in gut and cardiovascular disorders.

**Methods:**

Isolated tissue preparations such as, rabbit jejunum, rat and rabbit aorta and guinea pig atria were used to test the antispasmodic and cardiovascular relaxant effects and the possible mode of action(s) of the 70% aqueous-ethanolic extract of *Morinda citrifolia *roots (Mc.Cr).

**Results:**

The Mc.Cr produced a concentration-dependent relaxation of spontaneous and high K^+ ^induced contractions in isolated rabbit jejunum preparations. It also caused right ward shift in the concentration response curves of Ca^++^, similar to that of verapamil. In guinea-pig right atria, Mc.Cr caused inhibition of both atrial force and rate of spontaneous contractions. In rabbit thoracic aortic preparations, Mc.Cr also suppressed contractions induced by phenylephrine (1.0 μM) in normal- Ca^++ ^and Ca^++^-free Kerb's solutions and by high K^+^, similar to that of verapamil. In rat thoracic aortic preparations, Mc.Cr also relaxed the phenylephrine (1.0 μM)-induced contractions. The vasodilatory responses were not altered in the presence of L-NAME (0.1 mM) or atropine (1.0 μM) and removal of endothelium.

**Conclusions:**

These results suggest that the spasmolytic and vasodilator effects of Mc.Cr root extract are mediated possibly through blockade of voltage-dependent calcium channels and release of intracellular calcium, which may explain the medicinal use of *Morinda citrifolia *in diarrhea and hypertension. However, more detailed studies are required to assess the safety and efficacy of this plant.

## Background

*Morinda citrifolia *Linn (Fam. Rubiaceae) is commonly known as Noni or Indian mulberry. It occurs from India through Southeast Asia and Australia to Eastern Polynesia and Hawaii [1]. It is an edible plant and has been used in herbal remedies to treat various common diseases and to maintain overall good health [2]. Different parts of the plant including stem, bark, root, leaf, and fruits have been used in the system of traditional medicine to treat a broad range of diseases, including hypertension [3], atherosclerosis [2], colic [4] and diarrhea [5].

*Morinda citrifolia*, has been reported to possess antithrombotic [6], antioxidant [7], analgesic and antiinflammatory [8] and xanthine oxidase inhibitory [9] activities There are also preliminary studies reporting its blood pressure lowering[10] and vasodilatory [11] properties. However, the possible modes of action(s) of cardiovascular activities are lacking. The current study reports possible mode of action(s) of antihypertensive, vasodilator and antispasmodic activities.

## Methods

### Plant material

The vacuum dried 70% aqueous-ethanolic extract of *Morinda citrifolia *root was obtained from the Sami Labs Limited 19/1, 19/2, 1st Main II Phase, Peenya, Peenya Industrial Area Bangalor-560 058, India. The extract was prepared by following procedure as described by the manufacturer. Dried roots of *Morinda citrifolia *were procured from a reputed herb supplier in Southern India. The roots were chopped and ground by hammer mill and passed through 20 mesh screen. The powder (12 Kg) was extracted with 70% ethanol (48 L) at 70°C for 3 hours and filtered. The procedure repeated 2 more times and all filtrates combined and evaporated under vacuum. The dried extract was packed in polythene bags with nitrogen purge. The yield of the extract was 3.75%.

A sample of the extract was deposited at the herbarium of department of biological and biomedical sciences, Aga Khan University Karachi, Pakistan with voucher # MC-RT-02-08-82.

### Phytochemical screening

The *Morinda citrifolia *root extract was tested for the presence of various phytochemical classes of compounds such as alkaloids, phenolic compounds, strerols, flavonoids, tannins, coumarins and anthraquinones using method described by Khan and Gilani [12].

### Drugs and standards

The following reference chemicals were obtained from the source specified acetylcholine chloride, atropine sulfate, Nù-nitro-L-arginine methyl ester (L-NAME), phenylephrine hydrochloride, and verapamil hydrochloride (Sigma Chemical Company, ST Louis, MO). The following chemicals were used to make the physiological salts solution: potassium chloride (Sigma Chemicals Co.), calcium chloride, glucose, magnesium chloride, magnesium sulfate, potassium dihydrogen phosphate, sodium bicarbonate, sodium dihydrogen phosphate (Merck, Darmstadt, Germany) and sodium chloride (BDH Laboratory Supplies, Poole, England). All chemicals used were of the analytical grade available and dissolved in distilled water.

### Animals

Rabbits (1000-1300 g), guinea-pigs (500-600 g), Sprague-Dawley rats (180-220 g) and mice (20-25) of either sex were obtained from the animal house of the Aga Khan University, Karachi. The animals were housed in constant room temperature (23-25°C) and given free access to food and water. Rabbits and guinea-pigs were starved for 24 hrs prior to experiment and sacrificed by cervical dislocation. Experiments performed complied with the rulings of the Institute of Laboratory Animal Resources, Commission on Life Sciences [13] and approved by the Ethical Committee of the Aga Khan University, Karachi.

## Isolated tissue preparations

### Rabbit jejunum

The spasmolytic activity and possible mode of action of the plant materials were studied by using isolated rabbit jejunum as described previously [14]. Each segment of about 2 cm length was suspended in a 10 ml tissue bath containing Tyrode's solution, maintained at 37°C and aerated with a mixture of 95% oxygen and 5% carbon dioxide (carbogen). The tissues were allowed to equilibrate for at least 30 min at preload of 1 g. Tension changes in the tissue were recorded via a force displacement transducer (model FT-03) coupled with Bridge Amp and PowerLab 4/25 data acquisition system connected to computer running chart 5.3 software (ADInstrument, Sydney, Australia). The smooth muscles relaxant action of test materials was observed by administration of test drugs in a cumulative fashion directly without the use of an agonist.

To assess whether the spasmolytic activity of the test substance was through calcium channel blockade (CCB), high K^+ ^(80 mM), as KCl, was used to depolarize the preparations [15]. The K^+ ^(80 mM) was added to the tissue bath, which produced a sustained contraction. The test materials were then added in a cumulative fashion to obtain concentration-dependent inhibitory responses. To confirm the Ca^++ ^antagonist property of the test substance, the tissue was allowed to stabilize in normal Tyrode's solution, which was then replaced with Ca^++^-free Tyrode's solution containing EDTA (0.1 mM) for 30 minutes in order to remove Ca^++ ^from the tissues. This solution was further replaced with K^+^-rich and Ca^++^-free Tyrode's solution. Following an incubation period of 30 min, control concentration-response curves (CRCs) of Ca^++ ^were obtained. When the control Ca^++ ^CRCs were found super-imposable (usually after two cycles), the tissue was pre-treated with the crude extract for 60 min. The CRCs of Ca^++ ^were reconstructed in the presence of different concentrations of the extract.

### Rabbit and rat aortic preparations

Rabbit and rat aorte are used commonly to study the possible vasodilator effects of test drugs. Descending thoracic aorta, isolated from either rabbits or rat were obtained and cut into 2-3-mm wide rings that were individually mounted in 10 ml tissue bath containing Kerb's solutions maintained at 37°C and aerated with carbogen gas. A resting tension of 2 g was applied to each tissue changes in tension were recorded through a force displacement transducer (Fort-100, WPI, Hertfordshire, UK) couple to a bridge amplifier (Transbridge TMB4M, WPI) and PowerLab ML845 data acquisition system (ADInstrument, Sydney, Australia). An equilibrium period of one hour was allowed before experimentation with drug as described previously [16].

Rabbit aorta was used primarily to evaluate the effect of extract on K^+^- and PE-induced contraction to test if the extract has effect on voltage-operated and/or receptor-operated channels and on release of calcium from intracellular stores. Rat aorta was primarily used to further evaluate if the vasodilation caused by extract was endothelial-dependent or -independent [17]. Furthermore, using different species are helpful to see the species selective effects of any extract as described previously [18].

### Guinea-pig atria

Right atria isolated from guinea-pig killed by cervical dislocation were mounted in 20 ml tissue baths containing normal Kreb's solution maintained at 32°C and aerated with carbogen, as described previously [19]. The tissues were allowed to beat spontaneously under the resting tension of 1 g. Tension changes in the tissue were recorded through force-displacement transducer (model FT-03) using a grass Model 7 Polygraph. After equilibrium period of 30 minutes, control responses of isoprenaline (1 μM) and acetylcholine (1 μM) were obtained at least in duplicate. The test substances were then added cumulatively and the effect on force and rate of contractions was determined as percent of the pre-treated control.

### Acute toxicity test

Animal were divided in groups of 5 mice each. The test was performed using increasing doses of the plant extracts (3, 5, 10 g/kg), given orally, in 10 ml/kg volume to different groups serving as test groups. Another group of mice was administered saline (10 ml/kg, p.o.) served as negative control. The mice were allowed food and water *ad libitum *during a 24 hr test period and kept under regular observation for gross behavioural changes and mortality.

### Data analysis and statistics

All the data expressed as mean ± SEM and the median effective concentrations (EC_50_) values are given as geometric mean with 95% confidence intervals (CI). CRCs were analyzed by nonlinear regression (Sigmoidal dose-response curve variable slop). While One-way Analysis of Variance (one-way ANOVA) followed by Tukey's post-test was used to determine the significant difference in various doses, P value < 0.05 (P < 0.05) were considered statistically significant. All the graphs, calculation and statistical analyses were performed using GraphPad Prism software version 4.00 for Windows, (GraphPad Software, San Diego California USA, http://www.graphpad.com).

## Results

### Phytochemical screening

Qualitative phytochemical study that the *Morinda citrifolia *root extract show the presence of saponins, flavonoids, anthraquinine coumarines, sterols and phenolic compounds.

### Effects on rabbit jejunum

**Figure 1:**
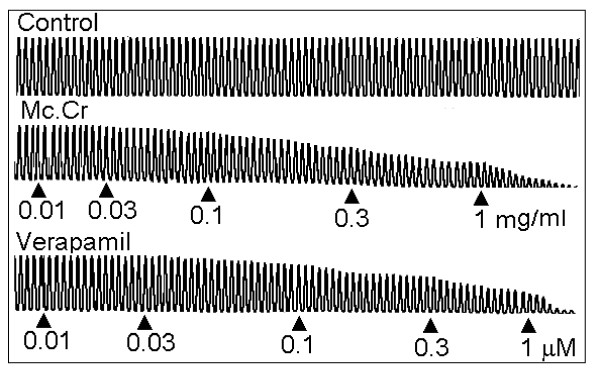
**Typical tracing showing inhibitory effect of crude extract of *Morinda citrifolia *(Mc.Cr) and verapamil on the spontaneous contractions in isolated rabbit jejunum preparations**.

**Figure 2:**
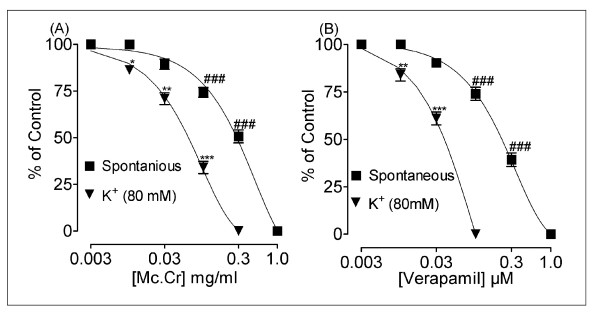
Inhibitory effect of the (A) Mc.Cr and (B) verapamil on spontaneous and high K^+^-induced contractions in isolated rabbit jejunum preparations ^###^P < 0.001; shows a comparison of concentration-dependent effects (specified effect compared with the effect of preceding dose) on spontaneous contractions, whereas **P < 0.01; ***P < 0.001; show comparisons of relaxant effects on K^+ ^(80 mM)-induced contractions.

**Figure 3:**
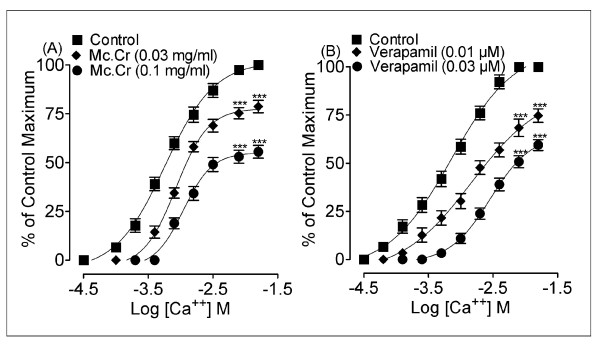
**Concentration-response curves of Ca^++ ^in the absence and presence of the increasing concentrations of Mc.Cr (A) and verapamil (B), constructed in Ca^++^-free and K^+^-rich (80 mM) Tyrode's solution in isolated rabbit jejunum**.***P < 0.001; compared to the respective control.

In isolated rabbit jejunum preparation, the root extract of *Morinda citrifolia *(Mc.Cr), inhibited the spontaneous contractions in a concentration-dependent manner with EC_50 _value (95% CI;) of 0.30 mg/ml (0.24 - 0.39; n = 4), similar to that of verapamil as shown in Figure 1. When tested against high K^+ ^(80 mM)-induced contractions, Mc.Cr caused relaxation with EC_50 _value of 0.06 mg/ml (0.05-0.08; n = 4) as shown in Figure 2A. Mc.Cr caused relaxation of K^+ ^(80 mM)-induced contractions at lower concentration compared to that of spontaneous contractions. Similar pattern was seen with verapamil, which produced relaxation of K^+ ^(80 mM)-induced contractions with EC_50 _value of 0.04 μM/ml (0.03-0.04; n = 4) at lower concentration than that of spontaneous contractions in rabbit jejunum with EC_50 _value of 0.23 μM/ml (0.19-0.27; n = 4) as shown in Figure 2B. Pre-treatment of the tissues with Mc.Cr (0.03 or 0.1 mg/ml; n = 4) showed rightward shift in the Ca^++ ^concentration response curves similar to that of verapamil as shown in Figure 3.

### Effect on thoracic aorta

**Figure 4:**
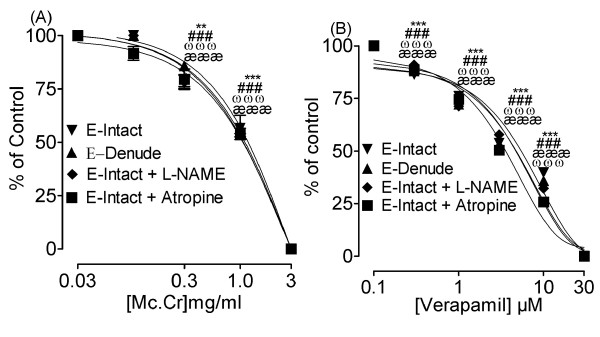
**Concentration-response curves showing the vasodilator effect of Mc.Cr (A) and verapamil (B) in the absence and presence of the N_ω_-nitro-l-arginine methyl ester (L-NAME, 0.1 mM) and atropine (1 μM) and on phenylephrine (PE 1 μM) induced contractions in isolated endothelium-intact and denude rat aorta preparations**. **P < 0.01; ***P < 0.001; shows a comparison of concentration-dependent effects(specified effect compared with the effect of preceding dose) on endothelial intact rat aorta, whereas, ^ωωω^P < 0.001; shows a comparison of concentration-dependent effect on endothelial denuded rat aorta and ^æææ^P < 0.001; shows a comparison of concentration-dependent effect on L-NAME with endothelial intact rat aorta, ^###^P < 0.001; shows a comparison of concentration-dependent effect on atropine pre-treatment with endothelial intact rat aorta.

In endothelium-intact rat aortic preparations precontracted with phenylepherine (PE 1 μM), Mc.Cr caused inhibition of induced contractions dose-dependently with EC_50 _value of 1.23 mg/ml (0.98-1.55; n = 4). The vasorelaxant activity was not altered in the presence of L-NAME (0.1 mM) with resulting EC_50 _value of 1.33 mg/ml (0.88-2.01; n = 4) or atropine (1.0 μM) with EC_50 _value of 1.30 mg/ml (0.85-1.97; n = 4), similarly removal of endothelium did not alter the potency. These effects were similar to those observed with verapamil as show in Figure 4.

**Figure 5:**
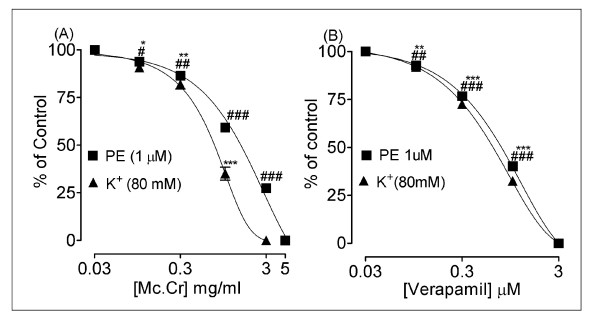
**Concentration-response curves showing the vasodilator effect of Mc.Cr (A) and verapamil (B) on phenylephrine (PE 1 μM) and K^+ ^(80 mM) induced contractions in isolated rabbit aortic preparations**. ^#^P < 0.05; ^##^P < 0.01; ^###^P < 0.001; shows a comparison of concentration-dependent effect on phenylephrine (PE 1 μM) induced contraction.*P < 0.05;**P < 0.01; ***P < 0.001; show comparisons of concentration dependent effects on K^+ ^(80 mM) induced contractions.

**Figure 6:**
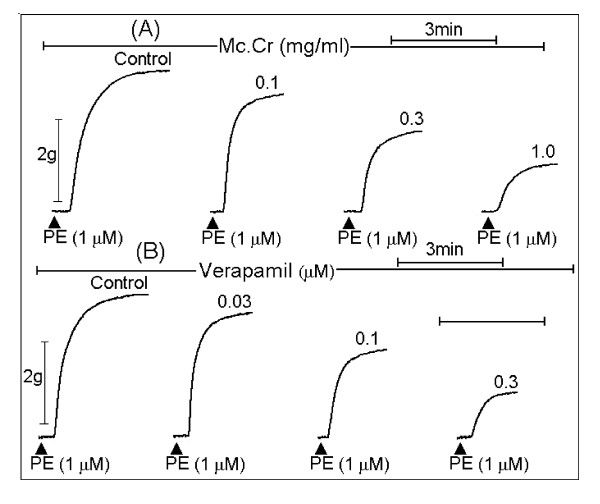
**Typical tracing showing the increasing concentration-dependent suppression of (A) Mc.Cr and (B) verapamil on control phenylephrine (PE 1 μM) peaks in the normal- Ca^++ ^and Ca^++^-free Kerb's solution**.

**Figure 7:**
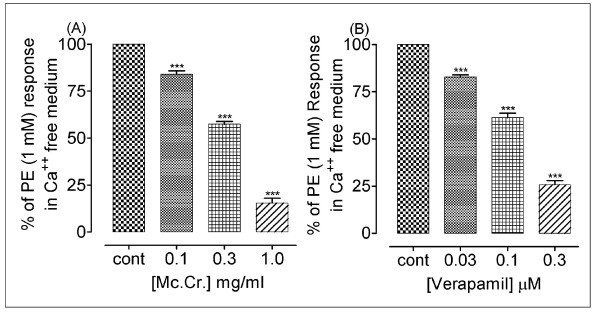
**Bar chart showing the inhibitory effects of (A) Mc.Cr and (B) verapamil on PE responses in Ca^++^-free Krebs solution in isolated rabbit aorta preparations**. ***P < 0.001; shows a comparison of dose dependent effect on phenylephrine (PE 1 μM) rabbit aorta.

In the rabbit aorta, Mc.Cr also caused concentration-dependent vasodilatation against PE (1 μM) and high K^+ ^(80 mM)-induced contractions, with EC_50 _value of 1.68 mg/ml (1.30-2.19; n = 4) and 0.76 mg/ml (0.69-0.84; n = 4), similar to that of verapamil as shown in Figure 5. Mc.Cr was also tested in increasing doses against PE (1 μM) peak responses as show in Figure 6, where it inhibited the PE (1 μM) peaks in normal-Ca^++ ^and Ca^++ ^free Kerb's solution dose-dependently (0.01-1.0 mg/ml, p < 0.001; n = 4), similar to that of verapamil (0.03-0.3 μM, n = 4) as shown in Figure 7.

### Effect on isolated guinea-pig atria

**Figure 8:**
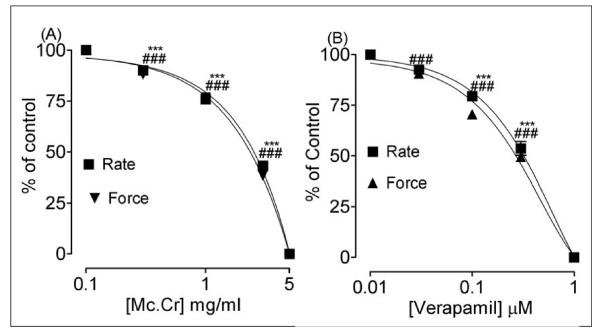
**Inhibitory effect of (A) Mc.Cr and (B) verapamil on the force and rate of spontaneous contractions of isolated guinea-pig atria**. ^#^P < 0.05; ^##^P < 0.01; ^###^P < 0.001; show comparison of concentration-dependent effects on force while *P < 0.05;**P < 0.01; ***P < 0.001; show comparison of concentration-dependent effects on rate of guinea-pig atria.

In isolated spontaneously beating guinea-pig atrial preparations, Mc.Cr exhibited concentration-dependent (0.03-5.0 mg/ml) suppression of the force and rate of contractions with EC_50 _value of 2.52 mg/ml (2.01-3.15; n = 4) and 2.69 mg/ml (2.05-3.53; n = 4) respectively. Similar effects of verapamil were seen in this preparation as shown in Figure 8. The inhibitory effects on heart were at relatively higher concentrations than those on smooth muscle preparations (P < 0.001).

### Acute toxicity

In mice, Mc.Cr did not cause any mortality and changes in gross behaviour up to the dose of 10 g/Kg as compared to control group.

## Discussion

The 70% aqueous-ethanolic extract of the *Morinda citrifolia *roots (Mc.Cr) caused a concentration-dependent inhibition of spontaneous contractions in isolated rabbit jejunum preparations, thus showing antispasmodic action. In earlier studies, we observed that the spasmolytic effect of medicinal plants is usually mediated through calcium channel blockade [20,21]. To assess whether the spasmolytic activity of this plant was also mediated through Ca^++ ^channel blockade (CCB), high K^+ ^(80 mM), as KCl, was used to depolarize the preparations [15]. High K^+ ^(> 30 mM) is known to cause smooth muscle contractions through opening of voltage-dependent L-type Ca^++ ^channels, thus allowing influx of extracellular Ca^++ ^causing a contractile effect [22] and a substance causing inhibition of high K^+^-induced contraction is considered as a blocker of Ca^++ ^influx [23]. Mc.Cr relaxed the high K^+ ^(80 mM)-induced contractions, similar to that caused by verapamil, a standard Ca^++ ^antagonist [24]. The presence of Ca^++ ^antagonist constituent(s) was further confirmed when pre-treatment of the tissue with Mc.Cr shifted the Ca^++ ^CRCs to the right. Calcium antagonists form an important therapeutic group and the common characteristic of these drugs are their dose-dependent inhibition of slow entry of Ca^++ ^and their capacity for reversal of this effect by Ca^++ ^[24]. The spasmolytic activity of Mc.Cr mediated possibly through Ca^++ ^antagonist effect explains its therapeutic usefulness in hyperactive gut disorders, such as abdominal colic and diarrhoea, as the CCB are known to be useful in such disorders [25].

Blood pressure is the product of cardiac output and peripheral resistance, increase in either or both can contribute to the development of hypertension. Since *Morinda citrifolia *is used in hypertension, the plant extract was studied further for its possible mode of action in isolated cardiovascular preparations. In guinea-pig right atria, Mc.Cr caused suppression of the force and rate of spontaneous contractions in a concentration-dependent manner, similar to that caused by verapamil. In rat and rabbit aortae, Mc.Cr also caused relaxation of contractions induced by either PE or high K^+^, similar to that of verapamil. In order to investigate if Mc.Cr also affects intracellular stores, Mc.Cr was tested against PE-induced contractions in Ca^++^-free solution, where it caused concentration-dependent suppression of contractile responses of PE, similar to those observed with verapamil, indicating that the extract is inhibiting the release of calcium from intracellular stores [17]. To investigate involvement of other mechanisms, the rat aorta was used. In this preparation, the vasodilatory response was not altered by removal of endothelium and in the presence of either atropine (a standard muscarinic antagonist) [26] or L-NAME (a standard nitric oxide synthase inhibitor) [27]. These finding suggest that the vasodilatory response of Mc.Cr is mediated through inhibition of receptor and voltage operated calcium channels and inhibition of release of calcium from intracellular stores. However, release of vasoactive substances from endothelium, or nitric oxide synthase or stimulation muscaric receptors do not appear to play role in the vasodilaroy activity of Mc.Cr.

Furthermore, the CCB effects of Mc.Cr in the gut or vascular tissues were observed at lower concentrations than the effect in cardiac tissue, which indicates selective effect of this plant in smooth muscle preparations. This difference may be due to the heterogeneity of calcium channels in different tissues, as different CCBs exhibit selectivity for a specific organ system [28]. Therefore, studies at molecular and cellular level are suggested to evaluate exact mechanism of action(s).

In acute toxicity studies Mc.Cr did not cause any mortality upto 10 g/Kg dose, which is much higher than the routinely used dose. However, more detailed subacute and chronic toxicity studies are required to establish the safety of this commonly used plant.

## Conclusions

In summary, the findings suggest that Mc.Cr has antispasmodic, vasodilator and cardiodepressant activities which are mediated possibly through blockade of calcium channels as well as release of calcium from intracellular stores. These findings may explain the medicinal use of *Morinda citrifolia *in abdominal colic, diarrhoea and hypertension. However, more detailed studies are required to establish the safety, efficacy and active constituents of this plant.

## Competing interests

The authors declare that they have no competing interests.

## Authors' contributions

AHG identified the plant, raised funds, supervised the work and refined the manuscript for publication. SRM carried out the draft, experimental work, data collection and evaluation, literature search and manuscript preparation. JI and MY co-supervised the work and corrected the manuscript for publication. NA helped in study design, analysis of data and preparing draft manuscript. AK and NR helped in the experimental work, analysis of data and preparing draft manuscript. All authors read and approved the final manuscript for publication.

## Pre-publication history

The pre-publication history for this paper can be accessed here:

http://www.biomedcentral.com/1472-6882/10/2/prepub
